# Incremental value of cardiac magnetic resonance in the characterization of unselected patients referred to exclude arrhythmogenic right ventricular cardiomyopathy

**DOI:** 10.1186/1532-429X-11-S1-O62

**Published:** 2009-01-28

**Authors:** Alberto Roghi, Stefano Pedretti, Patrizia Pedrotti, Santo Dellegrottaglie

**Affiliations:** Department of Cardiology, Niguarda Ca'Granda Hospital, Milan, Italy

**Keywords:** Cardiac Magnetic Resonance, Arrhythmogenic Right Ventricular Cardiomyopathy, Ventricular Cardiomyopathy, Inversion Recovery Sequence, Cardiac Magnetic Resonance Study

## Introduction

Cardiac magnetic resonance (CMR) may be efficiently applied to recognize morphologic and functional aspects employed in the diagnosis of arrhythmogenic right ventricular cardiomyopathy (ARVC). However, limited information is available regarding the actual impact of CMR findings in reaching a diagnosis of ARVC in an unselected population of patients referred to a CMR laboratory.

## Purpose

Aim of the study was to evaluate the incremental value of CMR over preliminary clinical/instrumental data in characterizing a group of consecutive patients referred for suspected ARVC.

## Methods

From January 2006 to April 2008, a total of 91 consecutive patients (60% males; mean age, 35 ± 18 years, range 8–72 years) underwent CMR (1.5 T, Magnetom Avanto, Siemens) to exclude ARVC. In each patient, CMR imaging protocol applied at the ventricular level included the acquisition of images for the identification of: (1) regional and/or global systolic dysfunction and chamber dilation (steady-state free precession sequences); (2) myocardial areas of fatty infiltration (T1-weighted fast spin-echo sequences with and without fat-saturation); myocardial areas of edema/flogosis (T2-weighted short-tau inversion recovery sequences); myocardial areas of delayed post-contrast enhancement (gradient-echo inversion recovery sequences). All patients had been previously evaluated by resting electrocardiogram, exercise stress test, 24-h Holter electrocardiogram monitoring and transthoracic echocardiography. Major and minor criteria for ARVC diagnosis were defined according to the standard Task Force criteria.

## Results

The studied patients were referred for the CMR study mainly based on frequent premature ventricular contractions (52%) or morphologic/functional alterations of the right ventricle (29%). By considering pre-CMR clinical/instrumental data only, diagnosis of ARVC was already reached in 3 (3%) patients, while the majority of patients (n = 62, 68%) presented with a low pre-test probability of disease. Inclusion of CMR data allowed to reach a diagnosis for ARVC in 4 (4%) patients. In none of the patients in the subgroup at low pre-test probability, CMR information led to a diagnosis for ARVC. With the CMR protocol employed in this study, diagnosis other than ARVC were obtained in 17 patients (19%), including: acute or chronic myocarditis (n = 5); chronic myocardial infarction (n = 2); partial anomalous pulmonary venous return (n = 2); other diagnoses (n = 8).

## Conclusion

In an unselected population of patients with suspected ARVC, the proportion of cases with a confirmed diagnosis of ARVC is not significantly impacted by CMR information over preliminary clinical/instrumental data. In 19% of patients referred to exclude ARVC, CMR allows the identification of previously unrecognized conditions other than ARVC.

